# Genetic structure and diversity of the Chilean flat oyster *Ostrea chilensis* (Bivalvia: Ostreidae) along its natural distribution from natural beds subject to different fishing histories

**DOI:** 10.1590/1678-4685-GMB-2021-0214

**Published:** 2022-03-09

**Authors:** Jorge E. Toro, Pablo A. Oyarzún, Felipe E. Toledo, Jorge M. Navarro, Alex F. Illesca, Jonathan P.A. Gardner

**Affiliations:** 1 Universidad Austral de Chile, Instituto de Ciencias Marinas y Limnológicas (ICML), Facultad de Ciencias, Valdivia, Chile.; 2 Universidad Andres Bello, Centro de Investigación Marina Quintay (CIMARQ), Quintay, Chile.; 3 Victoria University of Wellington, School of Biological Sciences, Wellington, New Zealand.; 4 Centro FONDAP de Investigación de Ecosistemas Marinos de Altas Latitudes (IDEAL), Valdivia, Chile.

**Keywords:** Genetic structure, Chilean flat oyster, genetic diversity

## Abstract

*Ostrea chilensis* (Küster, 1844), the flat oyster, is native to Chile and New Zealand. In Chile, it occurs in a few natural beds, from the northern part of Chiloé Island (41 ºS) to the Guaitecas Archipelago (45 ºS). This bivalve is slow growing, broods its young, and has very limited dispersal potential. The *Ostrea chilensis* fishery has been over-exploited for a number of decades such that in some locations oysters no longer exist. The aim of this study was to study the genetic diversity of the Chilean flat oyster along its natural distribution to quantify the possible impact of the dredge fishery on wild populations. The genetic structure and diversity of *Ostrea chilensis* from six natural beds with different histories of fishing activity were estimated. Based on mitochondrial (Cytb) and nuclear (ITS1) DNA sequence variation, our results provide evidence that genetic diversity is different among populations with recent history of wild dredge fishery efforts. We discuss the possible causes of these results. Ultimately, such new information may be used to develop and apply new management measures to promote the sustainable use of this valuable marine resource.

## Introduction

In Chile, the bivalve mollusc *Ostrea chilensis* occurs in only a few natural beds, from Calbuco (41º 45’S) to the Guaitecas Archipielago (45º 10’S) (Toro and Chaparro, 1990). Although the flat oyster is considered to be an endemic resource in Chile, it also occurs in New Zealand (Jeffs *et al*., 1997; Ó Foighil *et al*., 1999). The flat oyster is a bivalve that has protandrous hermaphroditism with sexual alternation and incubation of its embryos (Gleisner, 1981; DiSalvo *et al*., 1983). In Chile, the flat oyster is now a high-demand product because of its excellent taste qualities, and its economic value has increased accordingly over the last few decades. This increase in value has produced a significant reduction in size and number of the few natural bed of this species, including localised population extinction on Yaldad, Chiloé Island (unpublished data of Jorge Toro) and a significant decrease in the size of natural beds at Pullinque, Chiloé Island (Fundación Chinquihue, 2010).

**Figure 1 -:**
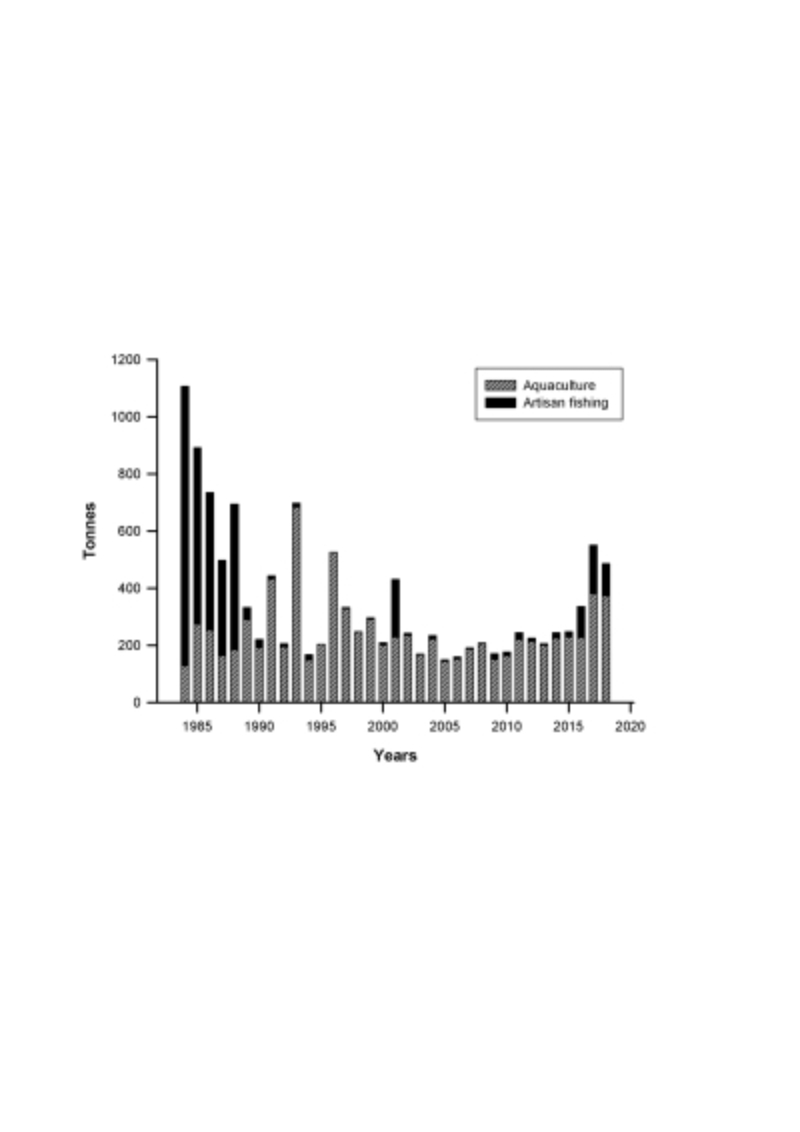
Exploitation (landings in tonnes) of the Chilean oyster (*Ostrea chilensis*) resource between 1984 and 2018.

Within its naturally limited spatial range in Chile, the oyster has been harvested for at least 77 years. As early as 1943, Pullinque, which is located in the interior area of the Gulf of Quetalmahue (Chiloé Island) was declared a Marine Reserve for the conservation of the flat oyster (SUBPESCA, 2004), due to the high fishery pressure on the natural beds of this species (Figure 1). Annually, following 2 to 3 weeks of incubation inside the pallial cavity, female oysters release larvae at the end of spring, usually in early December (DiSalvo *et al*., 1983). The larvae remain in the water column for a brief period (from a few minutes to hours - Millar and Hollis, 1963; Toro and Chaparro, 1990) and based on this short larval duration period are not expected to disperse very far from the parents, contributing to an expected low level of natural gene flow among populations (Buroker, 1985).

The flat oyster fishery has been over-exploited for about 4 decades (Lépez, 1983; López *et al*., 1988; Avila *et al*., 1996; see Figure 1), and its culture is not well developed, mainly because of its very slow growth rate (Toro and Chaparro, 1990; Toro and Newkirk, 1991). Because of the oyster’s slow growth rate (4-5 years to reach the market size - Toro and Newkirk, 1991) natural oyster beds are often exposed to illegal wild dredge fishing. Because of concerns about the state of oyster beds the Chilean Government set in place in 1954 management actions to regulate the oyster fishery, establishing an annual seasonal ban (from October 1^st^ to March 31^st^) with a minimun legal size for oysters (50 mm shell length - D.S. N°168-1985 SUBPESCA). Subsequently, after the start of *Ostrea chilensis* aquaculture in southern Chile, landings from the artisanal wild dredge fishery increased, because the prices of fished oysters were lower compared to aquaculture produced oysters. By 1984 fishery landings had reached 978 tonnes. However, because of the over-explotaition of the natural beds (López *et al*., 1988) by 1988 artisanal landings had decreased to < 20 tonnes per annum, although by 2016 this had increased to about 100 tonnes per annum (Figure 1). Despite this apparent recovery of the fishery to its earlier landing weights, a recent evaluation of the natural oyster bed at Pullinque (Chiloé Island) revealed that in only five years the over-exploitation of the wild dredge fishery has resulted in a reduction of 81% of oyster abundance (Fundación Chinquihue, 2010). As a consequence of this history of over-exploitation and reduction in population density, the genetic structure of flat oyster populations has probably been influenced both by the species’ unusual reproductive characteristics (i.e. low fecundity, larval brooding, limited dispersal potential) and the past and present histories of fishing activities (Toro and Chaparro, 1990; Toro and Gonzalez, 2009). In 2017, based on SUBPESCA Technical Reports, a permanent ban on all fishing activity was decreed for two years (D.E. N°768-2017 SUBPESCA) and renewed in 2020 for another 3 years (D.E No 32-2020), with the purpose of permitting the recovery of the natural beds at the northern area of Chiloé.

Effective management of an over-exploited species such as *Ostrea chilensis* requires an understanding of the species’ breeding system as well as its population genetic structure, effective population size, connectivity and genetic diversity (Buroker, 1985; Gaffney, 2006). In wild, non-exploited populations of many marine invertebrates the genetic diversity and effective population size are both expected to be very large because numbers of individuals (census size) are often huge (e.g., Hauser *et al*., 2002). However, over-exploitation in the form of extractive wild dredge fishing pressure, leading to a reduction in the number of individuals in a population, may contribute to lowered genetic diversity because reduced population sizes may in turn lead to increased inbreeding and subsequently to the fixation of deleterious alleles (Madsen *et al*., 1999; Charlesworth, 2003; Pinsky and Palumbi, 2014; Astorga *et al*., 2020). Ultimately, genetic diversity is directly relevant to population persistence because it is very closely connected with individual fitness (Frankham, 2005; Markert *et al*., 2010). A reduction in genetic diversity has been shown to reduce sperm quality (Borowsky *et al*., 2019), reduce survivorship of juveniles (Del Rio-Portilla and Beaumont, 2000), increase susceptibility to disease (Troncoso *et al*., 2000) and negatively effect physiological responses (Volckaert and Zouros, 1989; Zouros and Pogson, 1994; Launey and Hedgecock, 2001) across a range of bivalve species. 

One of the main problems that countries with fishery resources under exploitation have to deal with is the implementation of management measures to maintain stock size at a sustainable level over time (Gaffney, 2006; Beddington *et al*., 2007; Hare *et al*., 2011). Most fisheries are highly selective (i.e. by size) and this can cause a permanent change in the population’s size (age) structure. Therefore, any information regarding the genetic diversity and population genetic structure of a benthic fishery resource is valuable for the management of natural beds and may also contribute to aquaculture and the possibilty of wild stock enhancement (Allendorf *et al*., 2014; Ovenden *et al*., 2015; Casey *et al*., 2016). 

The aim of this study was to describe the genetic diversity of the Chilean flat oyster from sites along its natural distribution and to quantify the impact of the wild dredge fishery on the genetic diversity of the flat oyster. Finding a wild population that is not now fished is impossible, so testing for the impacts of fishing pressure on site-specific genetic diversity is very difficult. Because of this we compare genetic diversity amongst oysters from six sites (putative populations) with different histories of fishing pressure.

## Material and Methods

**Figure 2 -:**
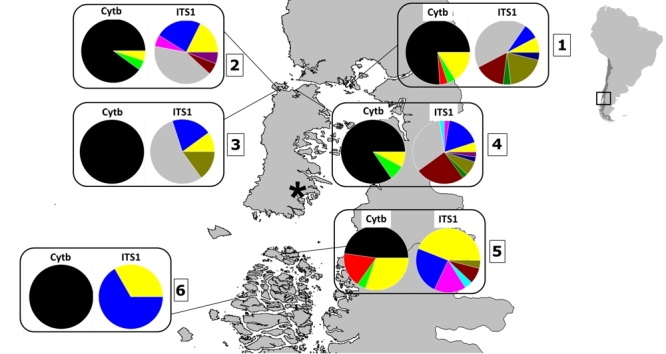
Distribution of haplogroups in *Ostrea chilensis* amongst the six sampling sites. Each colour represents a different haplotype (Cytb to the left and ITS1 to the right of each panel). Yellow = private haplotypes. 1 = Calbuco; 2 = Pullinque; 3 = Cayucan; 4 = Quempillén; 5 = Bahía Low; 6 = Isla Johnson. ^*^ Yaldad = No flat oysters (local extinction).

Samples of oysters were collected by dredging or diving from six sites that we subsequently refer to as wild source populations (Table 1), covering the whole range of the species’ natural distribution, from the north of Chiloé Island to the Guaitecas Archipielago in the south. Twenty-five to forty oysters from each sampled location (40.4 to 71.6 mm shell length), with a total of 165 oysters, were delivered live to the laboratory. The natural beds sampled (Figure 2) had different histories of exploitation: 1) Calbuco is the most northerly location and has natural oyster beds as well as several flat oyster aquaculture centres in the surrounding areas that use spat for aquaculture purposes collected from Pullinque. 2) Quempillén is a natural bed located in an estuary and is used mainly as a spat source for aquaculture centres with some management of the flat oyster reproductive stock (Ramirez, 2018). 3) Cayucan is a natural bed located close to Ancud city (Chiloé Island) near to, but outside, the Marine Reserve of Pullinque. 4) Pullinque is the Marine Reserve for the flat oyster and was used by the flat oyster aquaculture growers as a source of spat that were transported to other locations for grow out (i.e., this movement of spat represents human mediated gene flow). 5) Bahía Low is a natural bed located in the north side of the Guaitecas Archipelago, which is located in an area of permanent harmful algal blooms (HABs) (Diaz *et al*., 2014). 6) Isla Johnson is a very exposed location open to the Pacific Ocean with a reduced natural bed that is located south of the Guaitecas Archipelago and is surrounded by a few salmon aquaculture installations. It has no history of HABs. 

**Table 1 -:** Site survey information of sites along the Chilean coast (geographical coordinates of sites, number of *Ostrea chilensis* specimens collected (N), date of collection).

Site	Coordinates	N	Date
Calbuco	41°44’50.7’’S; 73°11’43.1’’W	25	21.01.2018
Quempillén	41°52’16.3’’S; 73°45’57.0’’W	40	26.01.2018
Cayucan	41°50’26.4’’S; 73°54’03.5’’W	25	06.09.2017
Pullinque	41°51’04.4’’S; 73°56’46.8’’W	25	05.12.2017
Bahía Low	43°50’03.3’’S; 73°54’55.7’’W	25	05.12.2017
Isla Johnson	44°21’27.4’’S; 74°24’1.76’’W	25	12.12.2017
Total number of oysters		165	

DNA extraction, PCR amplification, sequencing and alignment: Immediately after collection, a 1 cm^2^ piece of tissue was excised from the mantle border of each individual and was fixed in 95% ethanol and stored at 4 °C before DNA extraction. Total DNA was extracted using the genomic DNA mini-kit (Geneaid, New Taipei, Taiwan), according to manufacturer’s instructions. DNA samples were diluted 50-fold with ultrapure water, and PCR amplifications for the mitochondrial DNA cytochrome *b* (Cytb - 704 bp) and the nuclear DNA Internally Transcribed Spacer 1 (ITS1 - 494 bp) markers were performed using an Applied Biosystems® (Model Veriti) thermal cycler. For the amplification of the Cytb region, new primers were designed (Cytb Fost- F5’ TGT ATT CCC AGG TGG CTC TC 3’ and Cyt-*b* Rost -R5’ CTG CAC TCG CAT TCC TGA TA 3’). ITS1 amplification was performed using primers designed by Hedgecock *et al*. (1999) (ITS-1F- 5’ GGT TTC TGT AGG TGA ACC T 3’ and ITS-1-R 5’ CTG CGT TCT TCA TCG ACC C 3’). Amplifications were performed in a 25 µl reaction volume consisting of 2.5 µl 10x buffer (50 mM KCl, 10 mM Tris-HCl, pH 8.0), 1.0 µl of 50 mM MgCl_2_, 200 mM dNTPs, 0.5 µl of each primer (10 pg/µl), 1 U *Taq* (Invitrogen), 17.5 µl of double-distilled water plus 20 ng of DNA. Thermal cycling parameters for Cytb included an initial denaturation step at 95 °C for 3 min, followed by 30 cycles at 95 °C for 1 min, 54.4 °C for 1 min, and 72 °C for 1:30 min, and ended with a final 10 min extension at 72 °C. Thermal cycling parameters for ITS1 included an initial denaturation step at 95 °C for 3 min, followed by 30 cycles at 95 °C for 30 s, 60 °C for 20 s, and 72 °C for 30 s, and ended with a final 10 min extension at 72 °C. The samples did not exhibit double band amplifications as previously reported for this species by other authors (Mazón-Suástegui *et al*., 2016). All PCR products were scored on 2% agarose gels stained with SYBR® Safe DNA and photographed under a blue-light transilluminator (Invitrogen). For every gel, the size of the amplified fragments was estimated from a 100 bp DNA ladder (Invitrogen). Amplicons were purified and sequenced by Macrogen (Seoul, South Korea). Both sequence directions were determined, using the individual primers from the original reaction. DNA sequences were edited using Geneious® 11.0.4. (Biomatters Ltd, Auckland, New Zealand). All nucleotide sequences were aligned using MAFFT v.7 (Katoh and Standley, 2013) under the iterative method of global pairwise alignment (G-INS-i) (Katoh *et al*., 2005) and default settings were chosen for all parameters.

Standard diversity indices including number of haplotypes (K), number of segregating sites (S), haplotype diversity (H), mean number of pairwise differences (∏), and nucleotide diversity (π) were estimated for each population without regard to their fishing histories using DnaSP v.5.1 (Librado and Rozas, 2009). To measure deviation from the null hypothesis of constant population size and random mating, neutrality testing of Cytb and ITS1 sequence variation was conducted using the DnaSP software. Fu’s F_S_ (Fu, 1997) and Tajima’s D (Tajima, 1989) values were estimated by comparing the differences between the number of segregating sites and the average number of nucleotide differences for oysters from each site without regard to their fishing histories. Positive values indicate an absence of significant recent mutations that may have resulted from balancing selection, population structure or decline in population size. Negative values reflect excess recent mutations that may indicate population expansion or selective sweeps. The spatially explicit Bayesian clustering program Geneland 3.2.4 (Guillot *et al*., 2005), an extension program of R 3.1.2. (R Development Core Team, 2011), was used to investigate genetic structure. For concatenated (joined) Cytb and ITS1 sequence data, a multinomial distribution of genotypes conditionally based on allele frequencies, population membership and linkage equilibrium was assumed. We performed ten independent runs, where the parameters for possible populations were *K* = 1-6, with 5,000,000 MCMC iterations, saving every 100^th^ run. After comparing the results of the analyses, we selected a run with the highest posterior probability and post-processed it for graphical display. A burn-in of 10,000 generations (20%) was trimmed from the posterior in the post-processing. A contour map of the posterior mode of population membership was created to visualise the spatial genetic structure of the six populations without regard to their fishing histories. Past population dynamics in *Ostrea chilensis* was analysed using the Bayesian Skyline Plot in the program BEAST 1.8.1 (Drummond *et al*., 2012).

### Data Availability

The data that support the findings of this study are available on request from the corresponding author. The data are not publicly available due to Funding privacy restrictions.

## Results

Sequence data (Cytb = 704 bp; ITS1 = 494 bp) were obtained for 165 individuals from six populations of *Ostrea chilensis*. For Cytb the sequences were A-T rich (62.7%) compared to G-C content (37.3%). In contrast, for ITS1 the sequences were G-C rich (60.3%) compared to A-T content (39.7%).

Population genetic diversity: For Cytb, 11 nucleotide sites were polymorphic and 12 haplotypes were identified. For ITS-1, 13 nucleotide sites were polymorphic and 25 haplotypes were identified (Table 2). Haplotypic diversity was low for Cytb, but higher for ITS1 (Table 2). Despite sample sizes of n=25, two populations exhibited only one Cytb haplotype, although both had two or more ITS-1 haplotypes. For Cytb, one haplotype (H1) was found in every population and occurred at high frequency (82.6%) over the total data set (Figure 2). No other Cytb haplotype was shared by all locations. In total, 10.1% of the Cytb haplotypes were private (unique to a single population), most of them being singleton haplotypes (Figure 2). For ITS1, H6 (32.1%) was the most frequent haplotype. Only one ITS1 haplotype was shared by all locations (H4 = 19.1%) and for ITS1, 16% of the haplotypes were private. The population that showed the most private haplotypes was Bahía Low (Cytb = 30%; ITS1 = 44%) (Figure 2).

**Table 2 -:** Diversity indices and neutrality test results for *Ostrea chilensis* in southern Chile, based on data from Cytb and ITS sequence variation. K = number of haplotypes; H = haplotypic diversity; S = polymorphic sites; П = average number of pairwise differences; π = nucleotide diversity; Tajima’s *D* = Tajima’s *D* test; Fu’s *F*_*S*_ = Fu’s *F*_*S*_ test. Statistical significance: ^*^ = 0.05; ^**^ = 0.01. Cluster 1 from GENELAND analysis = Calbuco, Quempillén, Cayucan, Pullinque and Isla Johnson (Chile); Cluster 2 from the GENELAND analysis = Bahía Low (Chile). Ns = It was not possible to estimate.

Localities	K		H (SD)		S		Π		π		**Tajima’s *D***		**Fu’s *FS***	
	Cytb	ITS	Cytb	ITS	Cytb	ITS	Cytb	ITS	Cytb	ITS	Cytb	ITS	Cytb	ITS
Calbuco	6	8	0.427 (0.122)	0.770 (0.070)	7	7	0.633	1.307	0.00103	0.00277	-2.04^**^	-0.91	-3.38^*^	-3.26
Quempillén	5	16	0.283 (0.092)	0.924 (0.020)	4	8	0.300	1.982	0.00049	0.00423	-1.64	-0.18	-3.75^*^	-0.89
Cayucan	1	10	0.000 (0.000)	0.895 (0.043)	0	8	0.000	1.879	0.00000	0.00396	ns	-0.89	ns	-0.05
Pullinque	3	11	0.195 (0.115)	0.915 (0.050)	2	7	0.200	1.967	0.00032	0.00406	-1.51	-011	-1.86	-0.68
Isla Johnson	1	2	0.000 (0.000)	0.667 (0.314)	0	1	0.000	0.667	0.00000	0.00142	ns	ns	ns	ns
Bahía Low	5	11	0.700 (0.069)	0.897 (0.035)	4	10	0.957	3.640	0.00155	0.00755	-0.33	-1.23	-1.05	-2.08
Cluster 1	10	18	0.250 (0.056)	0.779 (0.031)	10	10	0.303	1.374	0.00049	0.00294	-2.22^**^	0.73	-12.91^*^	-13.10
Cluster 2	5	11	0.700 (0.069)	0.897 (0.035)	4	10	0.957	3.640	0.00155	0.00755	-0.33	-1.23	-1.05	-2.08
All locations	12	25	0.316 (0.050)	0.837 (0.021)	11	13	0.380	1.929	0.00062	0.00415	-2.07^*^	-0.50	-13.38^*^	-16.50

At the regional level, there was an apparent decrease in the total number of haplotypes from north to south, with 10 haplotypes (Cytb) and 18 haplotypes (ITS1) for cluster 1, and 5 haplotypes (Cytb) and 11 haplotypes (ITS1) for cluster 2 (Table 2). These groups relate to the proposed genetic structure (see below).

Differences in diversity indices were observed between the north and the south [Cluster 1 (Cytb): H = 0.250; **Π** = 0.303; **π** = 0.00049; Cluster 2: H = 0.700; **Π** = 0.957; **π** = 0.00155 / Cluster 1 (ITS1): **Π** = 1.374; **π** = 0.00294; Cluster 2: **Π** = 3.640; **π** = 0.00755 (see Table 2)]. These clusters correspond to those generated by Geneland (see below).

Demographic expansion: For Cytb and ITS1, for all six populations in all instances except one, Tajima’s *D* and Fu’s *F*_*S*_ values were negative, providing evidence of recent population expansion or selective sweeps (Table 2). When pooled across all populations, Tajima’s *D* and Fu’s *F*_*S*_ values were negative and significant (Table 2). 

The genetic differentiation between *Ostrea chilensis* populations based on Cytb (mtDNA) and ITS1 (nDNA) analysis, including their significant values, was carried out which gives a better understanding of the population structure (Table 3).

**Table 3 -:** Genetic differentiation between pairs of *Ostrea chilensis* populations based on Cytb (mtDNA) and ITS1 (nuclear DNA). G_ST_ (below diagonal) and N_ST_ (above diagonal) pairwise comparisons between the sites analysed from the South-eastern Pacific (southern Chile). Significant values (*P*<0.05) are indicated with an asterisk.

Cytb						
Locality	CA	QE	CY	PU	BL	JO
Calbuco (CA)		0.00611	0.00026	-0.00657	0.07972^*^	0.00462
Quempillén (QE)	-0.00042		0.00168	-0.01865	0.13083^*^	0.00531
Cayucan (CY)	0.09323^*^	0.04370		0.00000	0.14030^*^	0.00000
Pullique (PU)	0.00647	-0.02236	0.02632		0.11110^*^	0.00488
Bahía Low (BL)	0.10472^*^	0.19295^*^	0.31119^*^	0.20291^*^		0.14945^*^
Isla Johnson (JO)	0.10069	0.04843	0.00000	0.03228	0.32358^*^	
ITS1						
Localities	CA	QE	CY	PU	BL	JO
Calbuco (CA)		0.02963	0.01530	0.10883	0.12573^*^	-0.03363
Quempillén (QE)	0.00614		0.01672	0.00998	0.05436^*^	0.06555
Cayucan (CY)	-0.00714	0.04478		0.02434	0.10312^*^	0.03551
Pullique (PU)	0.00586	-0.01247	0.00275		0.06792^*^	0.18970
Bahía Low (BL)	0.13483^*^	0.08316^*^	0.17205^*^	0.07638^*^		0.05992
Isla Johnson (JO)	0.21813	0.13107	0.23364	0.09901	0.02583	

**Figure 3 -:**
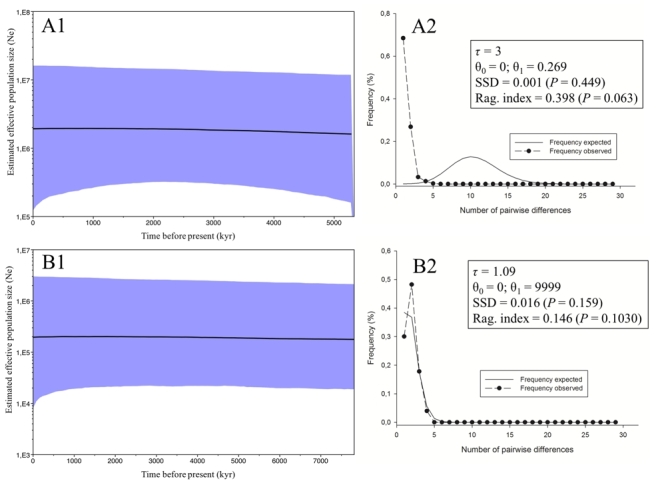
Bayesian skyline plot showing past demographic pattern for *Ostrea chilensis* for Cytb (A1 and ITS1 (B1). Black line indicates median estimates of population size, and the purple area indicates the upper and lower limits of the 95% confidence intervals. Mismatch distribution analyses for Cytb (A2) and ITS1 (B2) genes. τ time in generations since the last demographic expansion; *θ*_*0*_ initial population size; *θ*_*1*_ final population size; *SSD* sum of squared differences; *P* values in parentheses.

A Bayesian skyline plot, which shows the historical population dynamics for *Ostrea chilensis*, for Cytb (Figure 3 A1) and ITS1 (Figure 3 B1) revealed a pattern of population expansion. The mismatch distribution analysis for Cytb (Figure 3 A2) and ITS1 (Figure 3 B2) showed non-significant values for SSD and Raggedness indices; these results indicate that the null hyphotesis of demographic expansion cannot be rejected.

**Figure 4 -:**
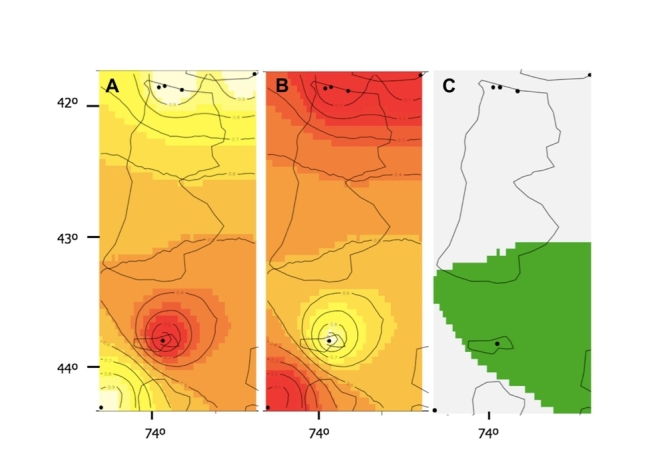
Geneland result for *K*=2 using the Geneland geospatial model with uncorrelated allele frequencies (data for Cytb and ITS1 sequence variation). A-B) plots representing the assignment of pixels to the Northern (A) and Southern (B) clusters of Chile; highest membership values are in light yellow, and the contour lines indicate the spatial position of genetic discontinuities between populations. C) Map of estimated posterior probability of population membership (by posterior mode) showing *K*=2 clusters for the grey area (north and far south) and for the green area.

Population genetic structure: GENELAND analysis of spatial population genetic structure based on concatenated Cytb+ITS1 sequence variation indicated *K* = 2 as the most likely number of clusters. The main group (Cluster 1) contained the 4 most northerly populations plus the most southerly, whilst the secondary group (Cluster 2) contained only the Bahía Low population. The assignment probabilities of individuals to their respective clusters were 0.90 (Figure 4).

## Discussion

Molecular-based genetic studies have become pivotal to help understand how over-fishing can affect the distribution, genetic structure and demography of populations, species and communities (e.g., Kenchington, 2001; Pérez-Ruzafa *et al*., 2006; Palero *et al*., 2011, Georgescu *et al*., 2015; Florescu *et al*., 2019). Bivalve molluscs such as oysters, which are ecological and economic components of coastal communities, have experienced an 85% reduction in biomass worldwide and 70% of natural oyster stocks are now in poor condition (Beck *et al*., 2011). This global decline has resulted in the implementation of restoration activities of natural beds, activities that must take into account the genetic diversity of the animals being used for restoration (Jaris *et al*., 2019).

Our results indicate that there are two main genetic groups of populations, one that includes the four locations in the northern part of Chiloé Island and also the most southerly oysters of Isla Johnson, and the other that is restricted to Bahía Low, located on Melinka Island (43º53’S). This evidence of a genetic discontinuity between Bahía Low and the other locations in this area is consistent with the known impacts of glacial cycles on Patagonian biota (Quaternary glaciations, especially the Last Glacial Maximum (LGM) 25-18 Ka - Hulton *et al*., 2002, McCulloch *et al*., 2000; Fraser *et al*., 2012). Traditional genetic models of glacial refugia and recolonisation routes have been proposed to describe the response of populations, species and communities to climatic changes (Provan and Bennett 2008; Zemlak *et al*., 2008, González-Wevar *et al*., 2012). It is proposed that species would have become restricted to glacial refugia outside the influence of glacial ice advances during cooling periods. After this, they expanded their distributions (Hewitt, 2004; Provan and Bennett, 2008). Therefore, unglaciated and refugial areas are expected to harbour higher levels of genetic diversity than peripheral, geologically altered, or newly founded regions. Bayesian skyline plots for Cytb and ITS1 showed the past population dynamics for *Ostrea chilensis*, with both markers revealing a pattern of population expansion. The mismatch distribution analysis for Cytb and ITS1 showed non-significant values for SSD and Raggedness indices; indicating the the null hyphothesis of demographic expansion cannot be rejected. Bahía Low is an area located on the northern side of Melinka Island: it is surrounded by small islands (i.e. Guacanec Island, Isla Martel, Islote Saturno, Isla Sargento, Isla Tinquinal, Isla Virginia, Isla Carril, Isla las Animas, Islote Pájaros Niños) that enclose the marine area. The same general area has been suggested as the most likely western refuge for other Patagonian taxa (e.g. *Galaxias platei* - Zemlak *et al*., 2008), perhaps within discontinuities of the ice field or on exposed portions of the Pacific continental shelf that was revealed by lowered sea levels. The high diversity and restricted distribution of the oyster haplotypes of Bahía Low, and signs of recent demographic expansion suggest that (1) this region was colonised during the glacial retreat and because it has not had significant population reduction, the oysters here have maintained high genetic diversity, or (2) Bahía Low was a glacial refugium for *O. chilensis* in the same way as proposed for other aquatic animals (Zemlak *et al*., 2008). 

Recently, there has been little influence of human activities (i.e., fishing) on the northern Guaitecas Archipielago (Melinka) *Ostrea chilensis* populations because of the almost permanent presence of harmful algal blooms (HABs) in this region (Lembeye, 2008; Diaz *et al*., 2014; Sandoval *et al*., 2018), that preclude the exploitation of oysters and other molluscs (e.g. mussels and clams). In the case of the Isla Johnson natural bed, which also showed low fishing pressure and reduced genetic diversity (see Tables 2 and 3), we hypothesise that there was a founder effect due to the transfer of juvenile spat from the Pullinque natural bed for oyster culture purposes (Toro and Chaparro, 1990, Litoral Austral, 2012). Also, there is a strong oceanographic current that separates Chiloé Island and the Guaitecas Archipielago that is located around 43°S, the West Wind Drift (WWD) (Strub *et al*., 1998) and the Corcovado superficial current (Silva *et al*., 1998), some or all of which may prevent the drifting of larvae between these two locations (i.e., there is a potential physical oceanographic barrier to gene flow here). 

Although pronounced genetic structure (i.e., high levels of regional genetic differentiation) are expected because *Ostrea chilensis* larvae have a short pelagic life (a few minutes to 10 hours - DiSalvo *et al*., 1983; Toro and Chaparro, 1990) and dispersal potential is expected to be low, our results indicate otherwise. The anthropogenic movement of juveniles (i.e. seed between 10-15 mm in size) from natural populations (e.g., Pullinque and Quempillén) to the oyster culture sites (Solis and Eberhard, 1979, Toro and Aguila, 1996; Toro and González, 2009, Litoral Austral, 2012) is likely to be the main cause of this higher than expected genetic similarity. It is likely that the genetic signature of the oysters that inhabit Isla Johnson may also be explained by the transfer of oyster seed from the Pullinque location to this site for aquaculture purposes (Litoral Austral, 2012).

As identified from Geneland, the *Ostrea chilensis* cluster that was composed of five sites exhibited reduced genetic diversity (by up to 64% for H, and 68% for ∏ and π) compared to the other cluster (one site only - Bahía Low) located on Melinka Island. These results may suggest that fishing pressure has contributed to changes in *Ostrea chilensis* genetic diversity, principally on those natural beds that experienced elevated fishing extraction pressure (i.e., populations located in the north of Chiloé - see Table 4). In addition, several other natural beds are now locally extinct, either due to artisanal fishing pressure (e.g., Yaldad and Castro, Chiloé island - see Figure 1) or to stochastic events (i.e. tsunamis) that have caused the sinking of the seabed (e.g. Carelmapu - continental Chile; Atwater *et al*., 2013). On the other hand, oyster farming in Chile is weakly regulated and policed by the authorities. Therefore, growers not only capture seed from the environment to grow them (for a period of 4-5 years) and they also extract wild oysters (wild fishery dredge) that are then sold as cultured oysters. Undoubtedly, there is a negative impact of these activities on the population gene pool and decreases in both population numbers and population sizes that are very difficult to estimate. This point is emphasised by the fact that there are no historical (i.e., before fishing began) data about genetic diversity and that it is now impossible to find a wild *Ostrea chilensis* population that has not been fished. Meaningful comparisons of genetic diversity between sites or populations (demes) are therefore very hard to make and the results are not as robust as we might like, but nonetheless, such comparisons are critically important if we are to understand how fishing pressure has impacted flat oyster genetic diversity and if improved management of the stock(s) is to take place in the immediate future.

**Table 4 -:** Artisanal fishing histories of the Chilean oyster (*Ostrea chilensis*) at the sites (and clusters) in southern Chile (2016-2017). Cluster 1 is located in the area where 96.3 % of the flat oyster cultivation activity is carried out.

Site	Artisanal Fishing - mean (SD)	Reference
Cluster 1		
Calbuco	46 (5.7) t.y^-1^	Sernapesca (2018)
Quempillén	2.05 (0.1) t.y^-1^	Ramirez (2018)
Cayucan	64 (17.0) t.y^-1^	Fundación Chinquihue (2010)
Pullinque	113.5 (58.7) t.y^-1^	Fundación Chinquihue (2010)
Isla Johnson	0.09 (0.01) t.y^-1^	Sernapesca (2018)
Total	140 (43.8) t.y^-1^	Sernapesca (2018)
Cluster 2		
Bahía Low	0.08 (0.01) t.y^-1^	Sernapesca (2018)

Genetic diversity has a fundamental role in the evolution of a species. Populations need a high level of genetic diversity to rapidly adapt to change or to stress (Barrett and Schluter, 2008). Reduced genetic diversity has been shown to decrease disease resistance (Spielman *et al*., 2004) as well as resilience to environmental disturbance (Hughes and Stachowicz, 2004). Those tasked with preserving the living natural resources should carefully consider how the overlap of aquaculture and wild populations will impact the genetic composition and evolutionary trajectories of populations. The reduction in population size of these natural beds should be taken into consideration in future management measures to recover the loss of genetic diversity. Future studies are necessary to understand how the loss of genetic diversity may be impacting oyster fitness and farming (e.g. growth rate or fertility) in the wild. In addition, genetic diversity from wild populations needs to be further monitored to ensure that no further reduction is allowed, and to maximise the diversity of the breeding pool for any hatchery-based production of seed that may occur in the future. Finally, we note that the application of only two DNA markers, one mitochondrial and one nuclear, does not capture the full extent of DNA variation in localised demes. The loss of site-specific genetic diversity for Cytb and ITS1 may be a reflection of still greater genetic loss throughout both genomes that is not apparent from our results, and which will lead to the loss of adaptation to localised environmental conditions. This problem is further exacerbated by the localised extinction of some flat oyster populations, which if true (i.e. if actually locally extinct as opposed to be functionally extinct) is of grave concern. Management considerations need to be developed that reflect these concerns to better manage the fishery before any further loss is experienced.
